# Sexual identity fluidity, identity management stress, and depression among sexual minority adolescents

**DOI:** 10.3389/fpsyg.2022.1075815

**Published:** 2023-01-11

**Authors:** Ankur Srivastava, William J. Hall, Evan A. Krueger, Jeremy T. Goldbach

**Affiliations:** ^1^School of Social Work, The University of North Carolina, Chapel Hill, NC, United States; ^2^School of Social Work, Tulane University, New Orleans, LA, United States; ^3^Brown School of Social Work, Washington University in St. Louis, St. Louis, MO, United States

**Keywords:** sexual minority adolescents, sexual identity fluidity, LGBTQ, mental health, identity management stress

## Abstract

**Introduction:**

Sexual identity is mutable and evolving, particularly during adolescence. Sexual identity fluidity could be stressful for some adolescents and may differ by birth-sex. Evidence suggests chronic stress can lead to negative mental health outcomes. However, it is unknown if these two processes (stress and depression) differ by sexual identity fluidity.

**Methods:**

This paper studied time-sequential associations between identity management stress and depression over time by sexual identity fluidity, in a national longitudinal data from sexual minority adolescents (SMA) aged 14–17 years using a multigroup autoregressive cross-lagged model (*n* = 1077).

**Results:**

In the sample, 40% of SMA reported at least one change in sexual identity over 18-month period. Greater number of cisgender females reported sexual identity fluidity compared to their male counterparts (46.9% vs. 26.6%). A temporal cross-lagged effect was reported between depression and identity management stress among cisgender females who reported fluidity in sexual identity; and no cross-lagged effect was reported among those females who did not report fluidity. However, among cisgender male sample depression predicted subsequent identity management stress, irrespective of their change sexual identity fluidity status.

**Conclusion:**

Public health programs and practice must be responsive to the sexual identity fluidity processes among adolescents, with particular attention to minority stress and depression. In addition, our results indicate that sexual identity development and fluidity processes differ between cisgender females and males; and the nuances associated with these processes of change need further investigation.

## Introduction

Adolescence is a time characterized by a greater reliance on peers, the onset of romantic relationships, and a strengthening sense of sexuality (Nickerson and Nagle, 2005; Brown and Bakken, 2011; Tolman and McClelland, 2011; Connolly et al., 2014). Adolescence is also a period when sexual identity begins to develop; though, experiences and processes of sexual identity development often differ among adolescents (Perrin, 2002; Savin-Williams and Cohen, 2004). For example, some adolescents may have a consistent sexual identity over time, whereas others may report shift in their sexual identity, referred as sexual identity fluidity (Ott et al., 2011; Katz-Wise and Hyde, 2015; Diamond et al., 2017). Sexual identity fluidity is more common among sexual minority adolescents (e.g., gay, lesbian, bisexual, queer), with recent estimates noting sexual identity changes among sexual minority youth ranging from 28% to 67% (Diamond, 2008; Morgan et al., 2018; Silva, 2018; Cohen et al., 2020; Srivastava et al., 2022).

Sexual identification can be stressful for youth, stemming from: social norms to maintain a consistent sexual identity over time; pressure to choose identity labels that may approximate heterosexuality (e.g., mostly heterosexual, bisexual, pansexual) due to the privileging of heterosexuality; and expectations to choose a label that may not fit with one’s dynamic experiences of sexual/romantic attractions, behaviors, and relationships (Hall, 2019). Some research suggests that sexual identity fluidity is associated with worse mental health, including depression (Everett, 2015; Feinstein et al., 2019).

There are several theories that may help explain the association between sexual identity fluidity and worsening mental health, including identity control theory and minority stress theory. Identity control theory posits that one’s identity must align with their internal assessment on how the identity and associated behaviors within the context of social structures where the identities are embedded (Burke, 2006). With respect to sexual identities, it would mean that different aspects of sexuality (i.e., attraction, behavior and identity) must have coherence and concordance and one is able to manage these within their social environments (Igartua et al., 2009; Rosario et al., 2011). However, discordance among these aspects of sexuality may result in one or more shift in sexual identity over time (Rosario et al., 2011). Applying the minority stress framework, the internal process of understanding, internalizing, and confusion in regard to one’s sexual identity may represent an additional sexual minority stressor, referred to as identity management stress (Goldbach et al., 2017). For example, discordance among aspects of sexuality or developing identity in a homonegative climate could lead to identity dissatisfaction, and stress (Igartua et al., 2009; Ott et al., 2011; Page et al., 2013; Horley and Clarke, 2016).

Decades of theoretical and empirical evidence have shown that chronic stress can lead to negative mental health outcomes, such as depression (Pearlin, 1999; Marin et al., 2011). Meyer (2003) argues that sexual minority adolescents are exposed to heightened stress related to a variety of stigma and discrimination-related experiences based on their non-heterosexual status, often referred to as sexual minority stress (Meyer, 2003; Marshal et al., 2011; Goldbach et al., 2014). Research suggests that adolescents experiencing stress associated with identity management (e.g., addressing disagreements between sexual behavior or attraction and sexual identification) may report more symptoms of depression (Clark et al., 2015; Everett, 2015; Caplan, 2017). Among sexual minority youth, depression occurs at a greater rate during adolescence, compared to their cisgender heterosexual peers (Cole et al., 2002). Unfortunately, both identity-related stressors and depression are also known to have harmful effects on multiple developmental outcomes (e.g., later mental health, educational and economic outcomes) through their late adolescence and early adulthood (Dekker et al., 2007; Fergusson et al., 2007; Bulhões et al., 2020).

In addition, these processes of sexual identity development may differ by sex assigned at birth. Some evidence suggests that cisgender males typically describe their sexuality often as unchanging; however, cisgender females often describe it as more fluid, evolving, and contextual, leading to different pathways of identity development (Kinnish et al., 2005; Diamond, 2008; Radtke, 2013). This difference in identity development by sex assigned at birth could be extended to differential rates of fluidity in sexuality identity between cisgender males and females. For example, research suggests that cisgender females may be more likely to report sexual identity fluidity compared to males over time (e.g., Oi and Wilkinson, 2018 [19.3 vs. 13.3%]; Fricke and Sironi, 2020 [18.0 vs. 6.2%], Savin-Williams et al., 2012 [17.8 vs. 6.2%], and Stewart et al., 2019 [26.0 vs. 11.0%]).

## Present study

Evidence suggests the importance of examining stressful experiences associated with identity management and depressive symptomology among sexual minority adolescents. However, these associations have not been studied longitudinally. In addition, it is unknown if these dynamic processes (identity management stress and depression) vary between those who report sexual identity fluidity versus those who did not. Moreover, given empirical evidence that sexual identity fluidity rates differ by sex assigned at birth, it is also important to understand if the association between identity management stress, depression, and sexual identity fluidity differs by sex assigned at birth.

The present study utilizes a large (*N* = 1,077) longitudinal sample of sexual minority adolescents. The goals of the present study are to assess time-sequential associations between two processes (identity management stress and depression) over time. In line with identity control theory, the present study sought to understand longitudinal associations between identity management stress and depression and how these processes may differ for those who report sexual identity fluidity versus those who do not.

## Methods

### Participants and procedures

A national community sample of sexual minority adolescents was recruited for a longitudinal investigation via targeted social media advertising (Facebook, Instagram, YouTube) based on geography and urbanicity/rurality to purposefully recruit adolescents from across the United States and in both urban and rural areas (2018-2022). A brief screener determined study eligibility (aged 14–17, identified as cisgender, provided a U.S.-based ZIP code, and reported a sexual attraction other than heterosexual or straight). Data come from a parent study (Schrager et al., 2022) of sexual minority adolescents aimed at understanding experiences of sexual minority stress and behavioral health during adolescence. To ensure data integrity, several checks for fraud (e.g., duplicate email address or contact information, screening out on first attempt and re-entering with false responses to get through the screener) and data quality (e.g., unrealistic survey completion times, low validation scores based on attention check measures, or decline to answer numerous questions) were completed before respondents were included in the finalized baseline data. Participants considered to be non-fraudulent were given the opportunity to refer up to three other adolescents into the study. All participants provided online assent prior to completing the survey. A total of 1,077 participants completed the baseline of the longitudinal investigation, and they were contacted for follow-up surveys every 6-months. The current analyses use 4 time-points (baseline, 6-months, 12-months, and 18-months follow-up). Participants received $15 for completing the baseline survey and could earn another $10 for each of the three people they referred to the study. Participants were paid incrementally for their participation in follow-up surveys. All study methods were approved by the authors’ University Institutional Review Board (masked for review).

## Measures

### Demographics

Demographic characteristics (age, race/ethnicity, sex at birth, sexual orientation, and socioeconomic status) were assessed with items created by the authors. The race/ethnicity item had six response options (Native American, American Indian, or Alaska Native; Asian or Pacific Islander; Black or African American; White; Latino or Hispanic; and race and ethnicity not listed); respondents could choose all categories with which they identified. Participants who chose multiple racial/ethnic categories were coded as multiracial. For analytic purposes, this variable was collapsed into six categories (White; Latino or Hispanic; multiracial or multiethnic; Black or African American; Asian or Pacific Islander; and Native American, American Indian, or Alaska Native). To assess sex, participants were asked “What was your sex assigned at birth?” Response options were “male” and “female.”

Sexual orientation/identity was assessed by asking an open-ended question, “What would you say is your sexual orientation or identity?” The research team used existing literature, prior work with sexual identity variables, and a range of responses on this question to design a qualitative coding scheme. The responses were coded as gay, lesbian, bisexual, pansexual, bisexual or pansexual, complex or multiple identities (e.g., gay pansexual, bisexual lesbian), queer, straight or mostly straight, asexual, and another identity (e.g., demisexual, agrosexual).

Sexual identity fluidity was measured as a reported change in sexual identity between waves. For the purpose of analysis, fluidity in identity was coded as 1 for reporting any change in sexual identity over 4 time-points and 0 for consistently reporting the same sexual identity across time-points.

### Identity management stress

To assess sexual identity-specific stress, we used the Identity Management subscale (α = 0.79; test–retest r = 0.90) from the 54-item Sexual Minority Adolescent Stress Inventory (Schrager et al., 2018). The subscale included 3 items (“I am questioning how to label my sexual orientation”; “I am having trouble accepting that I am LGBTQ.”; “I feel pressured to label myself as gay or lesbian.”); and responses to items were scored in a binary fashion: “Yes” responses are coded as 1, “No” responses are coded as 0. The subscale was administered for endorsement on items in the past 30 days at each time-point (baseline to 18-month follow up). The 30-day subscale scores are created as percentages of endorsed statements (i.e., 0, 33.3, 66.7, and 100.0%) (Goldbach et al., 2017).

### Depressive symptoms

Symptoms of depression were measured using the Center for Epidemiologic Studies Depression Scale Short Form (CES-D-4), which contains four items assessing the frequency of depression symptoms during the past week (“I felt depressed”; “I felt lonely”; “I had crying spells; “I felt sad”). Participants responded on a Likert scale with response options ranging from 0 (*rarely or none of the time [less than 1 day]*) to 3 (*most or all of the time [5–7 days]*); scores were summed (0–12; Melchior et al., 1993). Depressive symptoms were measured at every time-point, and internal consistency was high across time-points (Cronbach α = 0.83–0.85).

## Analytic plan

Bivariate analyses were used to examine the differences in reported sexual identity fluidity (ever versus no change) by demographic variables at baseline. T-test for difference in sample means were conducted for the continuous variable (age), while chi-square tests were conducted for categorical variables (sex assigned at birth, race/ethnicity and sexual identity).

**FIGURE 1:**
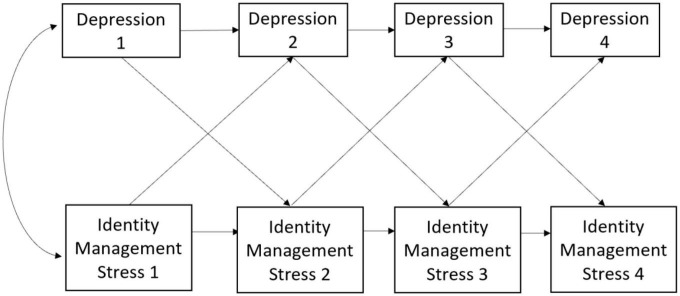
Time-sequential associations between depression symptoms and identity management.

We examined if there were differences in time-sequential associations between depression and identity management stress, by sexual identity change status (no change versus change) using a multigroup autoregressive cross-lagged (ARCL) model. The cross-lagged design comprises two or more variables at two or more time points. It yields three types of effects: synchronous associations (correlations between different variables measured at the same time), stability effects (correlations between the same variable measured at different times), and cross-lagged effects. The cross-lagged effects refer to the prediction of one or more variables by other (temporarily preceding) variables, controlling for the baseline level of the predicted variable. A multi-step process was employed to time-sequential associations between depression and identity management stress, by identity change status (Figure 1).

In our first model, each variable was allowed to predict subsequent follow-up assessment of itself, measuring the stability of individual differences in the construct from one occasion to the next. Cross-lagged effects were estimated, controlling for the previous level of the construct being predicted. Thus, when depression at 6-month follow-up was predicted by identity management stress at baseline, depression at baseline was controlled to rule out the possibility that the cross-lagged effect is simply due to correlations between depression and stress at baseline (Anyan et al., 2020). In our second model, we used a multiple group analysis framework where sexual identity change and sex assigned at birth variables were used to permit direct comparisons of the association between change in depressive symptoms and stress between groups. Model fit was determined using the following indices: χ2 goodness-of-fit statistic, the comparative fit index (CFI ≥ 0.90), the root-mean-square error of approximation (RMSEA ≤ 0.08), and the standardized root-mean-square residual (SRMR ≤ 0.08) (Browne and Cudeck, 1993; Hu and Bentler, 1999). Analyses were carried out in the structural equation modeling (SEM) framework using Mplus 8.0; We used the full information maximum likelihood estimator for all analyses, which assumes data are missing at random and uses all data available for each participant (Muthén and Muthén, 2009).

## Results

### Sample description

Table 1 contains demographic information. At baseline, the average age of the participants was 15.9 years (*SD* = 1.0); most reported sex assigned at birth as female (66.8%; n = 720). In terms of sexual identity, 38.8% (n = 418) identified as gay or lesbian, followed by bisexual (33.5%; n = 361), pansexual (12.4%; n = 133), bisexual or pansexual (4.0%; n = 43), complex or multiple identities (3.1%; n = 33), queer (2.7%; n = 29), questioning (1.7%; n = 18), asexual (1.6%; n = 16), mostly straight (1.3%; n = 14) and another identity (1.0%; *n* = 11). In the sample, 58.2% (n = 626) identified as White/Caucasian, followed by Latino/Hispanic (13.7%; n = 147), Multiracial (10.3%; n = 111), Black/African American (8.4%; n = 90), Asian/Pacific Islander (6.7%; n = 72), and Native American/America Indian (2.9%; n = 31).

**TABLE 1:** Participant characteristics (*N* = 1077; at baseline).

	*n* (%) or M (SD)	At least one change in sexual identity	No change in sexual identity	χ^2^ (df)
Age^#^	15.86 (0.98)	15.89 (0.97)	15.81 (0.98)	1.3634 (990)
**Sex assigned at birth^#^**				
Male	357 (33.2%)	84 (26.6%)	232 (73.4%)	
Female	720 (66.8%)	317 (46.9%)	359 (53.1%)	36.89 (1)*
**Race/Ethnicity^#^**				
White/Caucasian	626 (58.1%)	243 (42.1%)	334 (57.9%)	
Latino/Hispanic	147 (13.7%)	37 (28.2%)	94 (71.8%)	
Black or African American	90 (9.4%)	36 (44.4%)	45 (55.6%)	
Asian or Pacific Islander	72 (6.7%)	28 (40.0%)	42 (60.0%)	
Native American or American Indian	31 (2.9%)	15 (51.7%)	14 (48.3%)	
Multi-racial or multi-ethnic	111 (10.3%)	42 (40.4%)	62 (59.6%)	10.84 (5)
**Sexual identity^#^**				
Gay	239 (22.2%)	30 (14.0%)	184 (86.0%)	
Lesbian	179 (16.6%)	60 (36.8%)	103 (63.2%)	
Bisexual	361 (33.5%)	123 (35.9)	220 (64.1%)	
Pansexual	133 (12.4%)	64 (53.3%)	56 (46.7%)	
Bisexual or Pansexual	43 (4.0%)	37 (92.5%)	3 (7.5%)	
Multiple identities	33 (3.1%)	28 (87.5%)	4 (12.5%)	
Mostly straight	14 (1.3%)	9 (81.8%)	2 (18.2%)	
Queer	29 (2.7%)	17 (63.0%)	10 (37.0%)	
Questioning	18 (1.7%)	16 (100.0%)	0 (0.0%)	
Asexual	17 (1.6%)	9 (56.3%)	7 (43.7%)	
Another identity	11 (1.0%)	8 (80.0%)	2 (20.0%)	193.87 (10)*
**Change in sexual identity**				
At least one change	401 (40.4%)			
**Number of changes**				
0 times	591 (59.6%)			
1 time	228 (23.0%)			
2 or more times	173 (17.4%)			
**Identity management stress**				
Time 1	30.21 (32.24)	38.61 (32.9)	25.07 (30.5)	−6.65 (990)*
Time 2	26.69 (31.47)			
Time 3	26.87 (31.18)			
Time 4	26.95 (30.07)			
**Depression**				
Time 1	6.49 (3.40)	6.86 (3.32)	6.22 (3.43)	−2.93 (987)*
Time 2	6.27 (3.46)			
Time 3	5.90 (3.43)			
Time 4	6.02 (3.39)			

Age ranged from 14-17 years; Depressive symptoms ranged from 0 to 12; ^#^indicates values at Time 1; χ^2^ (df) = chi-square (degrees of freedom); χ^2^ (df) were provided for sex assigned at birth, race/ethnicity, and sexual identity; while *t*-test (degrees of freedom) values were provided for age, depression and identity management stress at baseline. * = significance at *p* value < 0.05.

### Sexual identity fluidity

In terms of sexual identity fluidity, around 40% of the sample (n = 401) reported at least one change in sexual identity over 4 time-points (18 months). In terms of number of changes, 23.0% (n = 228) reported one change, followed by two or more changes (17.4%; n = 173). In the sample, participants significantly differed in reported sexual identity fluidity (ever versus no change) by sex assigned at birth (26.6% cisgender males versus 49.6% cisgender females reported fluidity; χ^2^ (df) = 36.89 (1); p < 0.05) over the 18-month period. Similarly, participants also differed in reported sexual identity fluidity by sexual identity at baseline (χ^2^ (df) = 193.87 (10); p < 0.05). For example, 14% of those identified as gay at baseline reported a change in sexual identity over the 18-month period, compared to 36.8% lesbian, and 35.9% bisexual identified participants. These rates were found higher among those identified as pansexual (53.3%), bisexual or pansexual (92.5%), multiple identities (87.5%), mostly straight (81.8%), queer (63%), questioning (100%), and those identified with another identity (80.0%) at baseline. However, no significant differences were found in reported sexual identity fluidity by age and race/ethnicity (Table 1).

In addition, those who reported at least one change in sexual identity over 18-months, had significantly higher scores on identity management stress (38.61 vs. 25.07; *t*-test value = −6.65; *p* < 0.05) and depression (6.86 vs. 6.22; *t*-test value = −2.93; *p* < 0.05) at the baseline compared to those who did not report any change in sexual identity.

#### Depression and identity management stress

Results of our model fitting process indicated a model where effect of autoregressive components and cross-lagged were constrained to be equal over time in our multi-group ARCL models. Both our models resulted in adequate model fit (Model 1: grouping by sexual identity change status; CFI = 0.85, SRMR = 0.06, RMSEA = 0.08) (Model 2: grouping by sexual identity change status and sex assigned at birth; CFI = 0.84, SRMR = 0.07, RMSEA = 0.08) (Table 2). Although the comparative fit index (CFI) indicates minimally acceptable fit, the RMSEA and SRMR indicated that the models adequately fit the data (Browne and Cudeck, 1993; Hu and Bentler, 1999). Moreover, Lai and Green (2016) caution against strict cutoffs for data interpretation, stating that these two types of fit indices may diverge in the conclusions they suggest.

**TABLE 2:** Goodness of fit statistics for the mental health and minority stress ARCL models.

	df	χ^2^	*p*	CFI	SRMR	RMSEA
Model 1	124	474.14	*p* < 0.0001	0.85	0.06	0.075
Model 2	224	551.85	*p* < 0.0001	0.84	0.07	0.077

df, degrees of freedom; χ^2^, chi-square values; p, probability values; CFI, comparative fit index; SRMR, standardized root-mean-square residual; RMSEA, root mean square error approximation.

#### Cross-lagged effects

Model 1 examined the temporal association between identity management stress and depression by sexual identity fluidity status (Table 3). Results indicate depression predicted greater, subsequent, identity management stress for both groups, those who reported fluidity in sexual identity (β = 0.828; SE = 0.25; *p* < 0.001) and those who did not (β = 0.657; SE = 0.18; *p* = 0.001). However, identity management stress did not predict subsequent depression.

**TABLE 3:** Unstandardized estimates and standard errors for the ARCL Model 1 (grouping by sexual identity change).

	Model 1	
	No change	Change
	EST. (SE)	EST. (SE)
DEP (T2) ON STRESS (T1)	0.002 (0.002)	0.005 (0.003)
DEP (T3) ON STRESS (T2)	0.002 (0.002)	0.005 (0.003)
DEP (T4) ON STRESS (T3)	0.002 (0.002)	0.005 (0.003)
STRESS (T2) ON DEP (T1)	0.657 (0.18)*	0.828 (0.25)*
STRESS (T3) ON DEP (T2)	0.657 (0.18)*	0.828 (0.25)*
STRESS (T4) ON DEP (T3)	0.657 (0.18)*	0.828 (0.25)*
DEP (T2) ON DEP (T1)	0.582 (0.02)*	0.563 (0.03)*
DEP (T3) ON DEP (T2)	0.582 (0.02)*	0.563 (0.03)*
DEP (T4) ON DEP (T3)	0.582 (0.02)*	0.563 (0.03)*
STRESS (T2) ON STRESS (T1)	0.462 (0.02)*	0.432 (0.03)*
STRESS (T3) ON STRESS (T2)	0.462 (0.02)*	0.432 (0.03)*
STRESS (T4) ON STRESS (T3)	0.462 (0.02)*	0.432 (0.03)*

Models controlled for age, birth sex (reference: male) and race/ethnicity (reference: white). No Change, no change in sexual identity; Change, at least one change in sexual identity across 4 time points. DEP, depressive symptoms; STRESS, Identity Management Stress; T1-T4, time point 1- time point 4; EST, unstandardized estimates; SE, standard error; *= significance at *p* value < 0.05.

#### Difference by sex assigned at birth

Our final model examined the temporal association between identity management stress and depression by sexual identity fluidity status and sex assigned at birth (Table 4). Among cisgender females, those who reported fluidity in sexual identity, results indicated reciprocal associations between identity management stress and depression over time. Depression predicted greater, subsequent, identity management stress (β = 0.433; SE = 0.25; *p* < 0.05), and identity management stress predicted greater, subsequent, depression (β = 0.006; SE = 0.003; *p* < 0.05). However, among those cisgender females who did not report fluidity in sexual identity, we did not find any cross-lagged effects.

**TABLE 4:** Unstandardized estimates and standard errors for the ARCL Model 2 (grouping by sexual identity change status and sex assigned at birth).

	Model 2			
	Females		Males	
	No change	Change	No change	Change
	EST. (SE)	EST. (SE)	EST. (SE)	EST. (SE)
DEP (T2) ON STRESS (T1)	0.002 (0.003)	0.006 (0.003)*	−0.005 (0.004)	−0.002 (0.005)
DEP (T3) ON STRESS (T2)	0.002 (0.003)	0.006 (0.003)*	−0.005 (0.004)	−0.002 (0.005)
DEP (T4) ON STRESS (T3)	0.002 (0.003)	0.006 (0.003)*	−0.005 (0.004)	−0.002 (0.005)
STRESS (T2) ON DEP (T1)	0.433 (0.25)	0.638 (0.30)*	0.678 (0.27)*	1.173 (0.50)*
STRESS (T3) ON DEP (T2)	0.433 (0.25)	0.638 (0.30)*	0.678 (0.27)*	1.173 (0.50)*
STRESS (T4) ON DEP (T3)	0.433 (0.25)	0.638 (0.30)*	0.678 (0.27)*	1.173 (0.50)*
DEP (T2) ON DEP (T1)	0.571 (0.03)*	0.540 (0.03)*	0.509 (0.03)*	0.629 (0.05)*
DEP (T3) ON DEP (T2)	0.571 (0.03)*	0.540 (0.03)*	0.509 (0.03)*	0.629 (0.05)*
DEP (T4) ON DEP (T3)	0.571 (0.03)*	0.540 (0.03)*	0.509 (0.03)*	0.629 (0.05)*
STRESS (T2) ON STRESS (T1)	0.460 (0.03)*	0.436 (0.03)*	0.449 (0.03)*	0.411 (0.05)*
STRESS (T3) ON STRESS (T2)	0.460 (0.03)*	0.436 (0.03)*	0.449 (0.03)*	0.411 (0.05)*
STRESS (T4) ON STRESS (T3)	0.460 (0.03)*	0.436 (0.03)*	0.449 (0.03)*	0.411 (0.05)*

All the models controlled for age and race/ethnicity (reference: white). No Change, no change in sexual identity; Change, at least one change in sexual identity across 4 time points. DEP, depressive symptoms; STRESS, identity management stress; T1-T4, time point 1- time point 4; EST, unstandardized estimates; SE, standard error; *= significance at *p* value < 0.05.

Among cisgender males, results indicated that depression predicted greater, subsequent, identity management stress for both groups, those who reported fluidity in sexual identity (β = 1.173; SE = 0.50; *p* < 0.05) and those who did not (β = 0.678; SE = 0.27; *p* < 0.05). While, identity management stress did not predict subsequent depression in both cisgender male groups.

## Discussion

The current study advances our understanding of sexual identity fluidity, and its association with depression and identity management stress over time. The study examined difference in time-sequential associations between depression and identity management stress, by fluidity status using a multigroup autoregressive cross-lagged model. In the sample, 40% of sexual minority adolescents reported at least one change in sexual identity over 4 time-points over 18-month period. This finding is consistent with the literature, where 28-67% of sexual minority adolescents and youth have reported fluidity in sexual identity orientation over time (Diamond, 2008; Morgan et al., 2018; Silva, 2018; Cohen et al., 2020).

With regard to our research question (i.e., if temporal relationship between identity management stress and depression over time differs by sexual identity fluidity status), results show that depression predicted greater identity management stress, irrespective of sexual identity fluidity status. However, in our second model (grouping by fluidity in sexual identity and birth sex), results indicated that the association between depression and identity management stress over time differed by sexual identity fluidity for cisgender females, but not for cisgender males. Among cisgender females who reported fluidity in sexual identity, we found a reciprocal effect of identity management stress and depression over time (depression predicted subsequent identity management stress, and identity management stress predicted subsequent depression). However, among those females who did not report fluidity in sexual identity, we did not find any cross-lagged effects. Among males, we found that depression predicted subsequent identity management stress, irrespective of sexual identity fluidity status. This result draws support from limited evidence where adolescents with discordant or fluid sexual identity labels are more likely to report higher rates of depression (Everett, 2015; Caplan, 2017). For example, Caplan (2017) reported that in their school sample, those with a concordant sexual orientation (agreement between sexual behavior or attraction, and sexual identification) report significantly lower depressive symptoms scores than do those with a discordant sexual orientation. Our results suggest that among female adolescents, sexual identity fluidity is associated with cognitive and emotional disruptions as they reconfigure their identities and navigate social support networks, leading to heightened stress and depressive symptomology. Additionally, prior research examining impact of sexual identity fluidity on mental health risks have looked at change from the point of directionality (a change towards same-sex identities or a change towards heterosexual identities (e.g., Everett, 2015). However, in our study, we examined fluidity in sexual identity labels, and not in sexual orientation on a Likert scale. Hence, we are not able to assess directionality of change, as the understanding of authentic sexual identity and a shift toward or away from it would differ from individual to individual.

Our results indicate that sexual identity fluidity process differs between cisgender females and males. Among males, the association between these two processes (identity management stress and depression) did not differ by sexual identity fluidity status. The suggested difference in that sexual identity change process between cisgender females and males, could be attributed to difference in sexual identity development and related experiences by birth sex (Kinnish et al., 2005; Diamond, 2008; Radtke, 2013). A recent systematic review of changes in sexual identities by Srivastava et al. (2022) found that sexual identity fluidity was more common among cisgender female compared to their male counterparts (often by more than 10 percentage points); however, it remains difficult to draw reliable conclusions about the extent and cause of these differences by sex assigned at birth. We believe one of the reasons for higher rates of fluidity among females in our sample compared to males could be attributed to sexual identification at baseline. For example, in our sample, those who identified with less common sexual identities (ex., pansexual, asexual, queer, and questioning) at baseline were more likely to report a change in sexual identity over 18-month period, compared to those who identified as gay, lesbian, and bisexual. *Post hoc* analyses suggests that 85% of cisgender males identified with more common sexual identities (gay or bisexual) at baseline, compared to only 66% of cisgender females. Further, those identifying as more common identities (gay, lesbian, bisexual) were least likely to report a change at the subsequent time point (14.1% vs. 49.3%; χ^2^ = 132.1, *p* < 0.001) compared to participants identifying with less common identities (ex., pansexual, asexual, queer, and questioning). Given the variations in reported changes and reasons for the change, more research is needed to understand the causes of such differences by assigned sex at birth. Our finding also finds some support in the extant literature, for example, Glover et al. (2009) reported that in their study males used more traditional label (e.g., gay), whereas females presented greater variability in attraction and self-labels of sexual orientation, and were more likely to experience non-exclusive attractions, thus making sexual orientation and identification a much more fluid process compared to males. Some of these differences can be explained by dominant notions of masculinity for males and more acceptability of fluidity in sexuality for females (Kilianski, 2003; Breen and Karpinski, 2013; Diamond, 2016); however, the nuances associated with sexual identity development and processes of change as they differ between cisgender females and males need further investigation.

Current research and practice with sexual minority adolescents has suggested role of enabling environment in healthy identity development among sexual minority adolescents (Perrin, 2002; Savin-Williams and Cohen, 2004; Legate et al., 2019). Studies suggest that adolescents with unsupportive or homophobic familial environment, delay their coming out processes or even try to meet the heterosexual expectations of their predominately heterosexual families (Waldner and Magrader, 1999; Cox et al., 2010; Legate et al., 2019). For example, a study with 502 adolescents reported less acceptance and greater difficulty with disclosure were associated with higher scores on internalized homophobia (negative self-perceptions and self-devaluation for being non-heterosexual) (Cox et al., 2010). Additionally, confusion about one’s sexual identity can also delay endorsement and integration of sexual identity label (Igartua et al., 2009; Rosario et al., 2011). In short, lack of enabling environment may result in delaying of identity integration, resulting in development of less-authentic identities likely to change later. We believe some adolescents are more likely to report sexual identity fluidity than others, which may be because of underlying factors like access to community and support, relationship status, presence of a role model, etc. Moreover, these processes of fluidity could also be an additional stressor among sexual minority adolescents. The results add to the limited knowledge on the complex relationship between minority stress and depression as it relates to sexual identity fluidity.

## Limitations and conclusion

This study had several limitations. This study focused on cisgender youth, it does not discuss the disparities experienced by transgender and gender non-binary adolescents (Price-Feeney et al., 2020; Srivastava et al., 2020). Sexual identity fluidity were measured as a difference in response to sexual identity question between two time-points, however, this may not be a conscious change on adolescent’s part. Internet survey research has distinct advantages, especially for reaching marginalized, geographically dispersed minority populations (Stern et al., 2020). We recruited a large sample of diverse sexual minority adolescents from both urban and rural areas of United States. However, internet-based recruitment and data collection also have limitations and challenges. In terms of generalizability, our findings are limited to adolescents who have access to the internet and online spaces. Although there is now significant evidence that the demographic and behavioral characteristics of those recruited online are similar to those recruited through more traditional, in-person venues. Additionally, internet survey research also has validity concerns (e.g., duplicate participations). However, our study protocols addressed these concerns through rigorous validity checks. All data were self-reported; however, anonymity was ensured by not collecting any identifying information, which minimized response bias.

Despite these limitations, to our knowledge, this paper is the first to examine the difference in relationship between depression and identity management stress by sexual identity fluidity status in a nationwide sample of sexual minority adolescents. Given pervasive homonegative social and political climates in many areas, sexual minority adolescents will continue to experience sexual identity development in less supportive environments, adding to the minority stress experiences. Among females, the evidence of relationship between identity management stress and depression as it associates with sexual identity fluidity is important to support female adolescents through their healthy identity development processes. However, the underlying and understudied variations by sex and gender need further examination.

## Data availability statement

The raw data supporting the conclusions of this article will be made available by the authors, without undue reservation.

## Ethics statement

The studies involving human participants were reviewed and approved by University of Southern California Institutional Review Board. Written informed consent for participation was not provided by the participants’ legal guardians/next of kin because: All participants provided assent to participate, and longitudinal participants provided informed consent at the first follow-up survey after reaching age 18. All study procedures were reviewed and approved by the University of Southern California Social–Behavioral Institutional Review Board, including a waiver of parental permission given the potential for harm due to unintentional “outing” to a parent during the consent process.

## Author contributions

AS contributed to the conceptualization, formal analysis, and writing, reviewing, and editing the manuscript. WJH and EAK contributed to the interpretation of results and editing the manuscript. JTG is the PI on the grant, and contributed to the conceptualization, and interpretation of results. All authors contributed to the manuscript revision, read, and approved the submitted version.
